# A Systematic Assessment of Prevalence, Incidence and Regional Distribution of Multiple Sclerosis in Bavaria From 2006 to 2015

**DOI:** 10.3389/fneur.2018.00871

**Published:** 2018-10-30

**Authors:** Tanja Daltrozzo, Alexander Hapfelmeier, Ewan Donnachie, Antonius Schneider, Bernhard Hemmer

**Affiliations:** ^1^Department of Neurology, Klinikum Rechts der Isar, School of Medicine, Technical University of Munich, Munich, Germany; ^2^School of Medicine, Institute of Medical Informatics, Statistics and Epidemiology, Technical University of Munich, Munich, Germany; ^3^National Association of Statutory Health Insurance Physicians of Bavaria, Munich, Germany; ^4^Institute of General Practice School of Medicine, Technical University of Munich, Munich, Germany; ^5^Munich Cluster for Systems Neurology (SyNergy), Munich, Germany

**Keywords:** multiple sclerosis, prevalence, incidence, regional distribution, Bavaria, Germany

## Abstract

**Introduction:** Worldwide, incidence and prevalence of multiple sclerosis (MS) have increased over the last decades. We present a systematic epidemiological study with recent prevalence and incidence rates of MS in Bavaria.

**Methods:** Incidence and prevalence of MS stratified by gender, age groups and region were analyzed by data records from 2006 to 2015 of more than 10 million people insured by the *Bavarian Association of Statutory Health Insurance Physicians*. Official statistics of the *German Federal Ministry of Health* provided the size of the general population. Future prevalence was estimated with a predictive model.

**Results:** From 2006 to 2015 prevalence of MS in Bavaria increased from 171 per 100,000 to 277 per 100,000, while incidence rates remained relatively stable (range 16–18 per 100,000 inhabitants with a female to male ratio between 2.4:1 and 2:1). Incidence and prevalence were higher in urban than urbanized and rural areas. The prevalence is expected to increase to 374 per 100,000 in 2040 with the highest prevalence rates between 50 and 65 years.

**Conclusion:** The prevalence of MS in Bavaria is among the highest worldwide and will further rise over the next two decades. This demonstrates a need to strengthen healthcare provision systems due to the increasing numbers of particularly older patients with MS in the future.

## Introduction

Multiple sclerosis (MS) is one of the leading causes for disability in young adults with a major impact on quality of life of the affected persons (1, 2). Europe is a region with high prevalence rates with half of the persons reported to be affected by MS in the world living in Europe (3, 4). Population-based data from 1985 to 2011 suggest an increase of prevalence of MS worldwide with an increase of the overall number of people with MS from 2.1 million in 2008 to 2.3 million in 2013 according to the WHO (3–5). In population-based studies from 1985 to 2011, worldwide incidence rates of MS ranged from <1 per 100,000 to > 10 per 100,000 (4, 6, 7). The prevalence and incidence of MS is higher for women than men (ranging from 1.1:1 to 3:1 in the majority of European studies) (4). Over the last decades, prevalence and incidence grew at a faster rate in women than in men (4, 6–9).

In central Europe, including Switzerland, Germany, Austria and Hungary, prevalence rates ranged from 62 per 100,000 to 128 per 100,000 with the lowest rates found in the 1990s in Hungary and the highest prevalence rates analyzed in 2006 in Germany (4).

From 2005 to 2009 an increase of prevalence from 102 per 100,000 to 143 per 100,000 in Germany was reported (10). At the beginning of this century, the overall number of MS patients in Germany was estimated with approximately 120,000 to 140,000 patients and a female to male ratio of 2.5:1 (11, 12). Recent estimations of the health care provision system report about an overall number of MS patients in Germany of 200,000 (13). Reported incidence rates ranged from 4.6 per 100,000 in 1985 to 8.0 per 100,000 (14, 15). Due to the variety of data sources and survey methods applied to different geographic populations, the comparability of such results is inherently limited.

This project aimed to estimate the prevalence and incidence of MS in the German federal state of Bavaria in the years of 2006–2015 together with their regional distribution. The study comprises a systematic retrospective analysis of anonymous claims data held by the *Bavarian Association of Statutory Health Insurance Physicians*, covering about 85% of the population of Bavaria.

## Methods

The incidence, prevalence and corresponding regional distribution of MS in Bavaria were estimated on a yearly basis from two different data sources. Ambulatory claims data held by the *Bavarian Association of Statutory Health Insurance Physicians* (BASHIP) were used to assess the number of MS patients with a secured ICD-10 diagnosis G35. Study data, aggregated by year gender and age group were extracted from a pseudonymised database created by the BASHIP for the purpose of health services research. Approval was obtained from the responsible data protection officer of the BASHIP. Data covered the years 2004 to 2016 and was stratified by region, gender and the age groups [0, 15), [15, 20), [20, 25), …, [70, 110]. Here, the mathematical notation of intervals is used to indicate inclusion by squared brackets “[” and “]”, and exclusion by round brackets “(” and “)”. Therefore a patient transitions from the interval [0, 15) to the interval [15, 20) on the 15th birthday, for example. Estimates of incidence and prevalence have also been standardized to the age distribution of the European standard population (ESP) and the WHO standard population to support comparability with data of other countries (16, 17).

A patient was considered to have MS if the secured ICD-10 diagnosis G35 was present in at least two separate quarterly periods, not necessarily in the same year. The year of diagnosis was considered to be the year of incidence; a minimum period of 2.5 years was available by which to exclude a previous MS diagnosis. In subsequent years, the patient was only included in the prevalence count if a corresponding MS diagnosis was present. An MS diagnosis was assumed to be valid coded by a neurologist at least once during the observation period.

The underlying population was taken from the official KM 6-statistic of the *German Federal Ministry of Health*. This details the number of statutorily insured persons in Bavaria by age group and gender, but not by administrative district. The latter was therefore inferred from the regional distribution of the patient's districts of residence as observed in the BASHIP claims data.

Patients were also aggregated according to the regional planning districts as defined by the *German Federal Institute for Research on building, urban affairs and spatial development*, each containing between 317,000 and 2.8 million residents and classified using the categories urban, partially urbanized and rural. In urban areas, 50% of the population live in cities and the overall population density is >150 inhabitants per km^2^. In rural areas, less than 50% of the population live in cities and the population density outside the cities is <100 inhabitants per km^2^. Intermediate regions are classed as partially urbanized.

Data were restricted to the years 2006 until 2015. In an additional analysis, the prevalence for the period 2020–2040 was forecast starting with the actual data for the year 2015. For each discrete 5-year interval, the count of MS patients and the size of the general population in 2015 were transitioned from one age group to the next. At each step, several factors including the MS incidence affect the group sizes either positively or negatively. The mean values of the age specific incidence rates, which proved to be rather stable within the years 2006–2015, were used for this computation. Assuming a uniform distribution of age within the age groups, each subject spends an average of 2.5 years under the risk of two subsequent age groups considering a time interval of 5 years. The corresponding incidence rates where therefore each multiplied by a factor of 2.5 and applied to the size of the general population of a given age group to add the resulting expected count of new MS diagnoses in the transition of this age group to the next age group for a step of 5 years. In the same manner and taking the same effect, one-third of the MS patients and the general population in age group [0, 15) transitioned to the next age group. The size of the age group [0, 15) was kept stable in this analysis, which assumes constant birth rates. This stable amount of subjects not transitioning into the next age group stayed under a stable risk estimated for this age group [0, 15) for five years in each step. There was also no transition of patients from the age group [70,110] to any older age group and subjects stayed under the risk of this age group, i.e., applying the corresponding incidence, for a period of 5 years in each step. Taking a negative effect on the count of MS patients, e.g., due to death, movements or any other cause, dropout rates were computed for each age group and year from the BASHIP claims data by identification of MS patients with terminating records, i.e., patients without any claims records of any kind in the years after a given year. These dropout rates were rather stable and were averaged across years for each age group and gender. In the transition of MS patients from one age group to the other, i.e., for a time shift of 5 years, the average dropout rate of two subsequent age groups was applied five times to resemble yearly dropout. Further losses and gains also had to be expected for any cause, such as death and population movement, in the general population. Annual dropout and accrual rates between 2013 and 2014 stratified by age group and gender were therefore estimated using the so-called “birth day sample” (German: *Geburtstagsstichprobe*) of anonymous claims data that has been augmented with a list of all insured persons and is available from the german *Institut des Bewertungsausschusses* (18). These rates were used to adjust the size of the general population in the forecast as described for the dropout of MS patients above. Concerning the oldest age group [70,110] it is evident that dropout, most likely due to death, has to be taken into account as otherwise, this group size would constantly rise due to subjects transitioning into this age group with each step of 5 years taken in the forecast.

All computations were performed in R 3.4.2 (R Foundation for Statistical Computing, Vienna, Austria).

## Results

### General aspects

Of the 12.8 million inhabitants of Bavaria in 2015, approximately 83.5% were covered by statutory health insurance plans (*Federal Statistical Office, 2015*). Characteristics of the populations of statutorily and privately insured persons can be inferred from the 2015 microcensus results provided by the Federal Statistical Office. Therefore the median age of statutorily and privately insured persons was 46 and 48 years, with 52.3 and 41.5% women and a median net income of 900–1300€ and 2000–2600€, respectively. Gender is a well-known risk factor for MS and has been taken into account in the following computations.

### Prevalence

**Figure 1:**
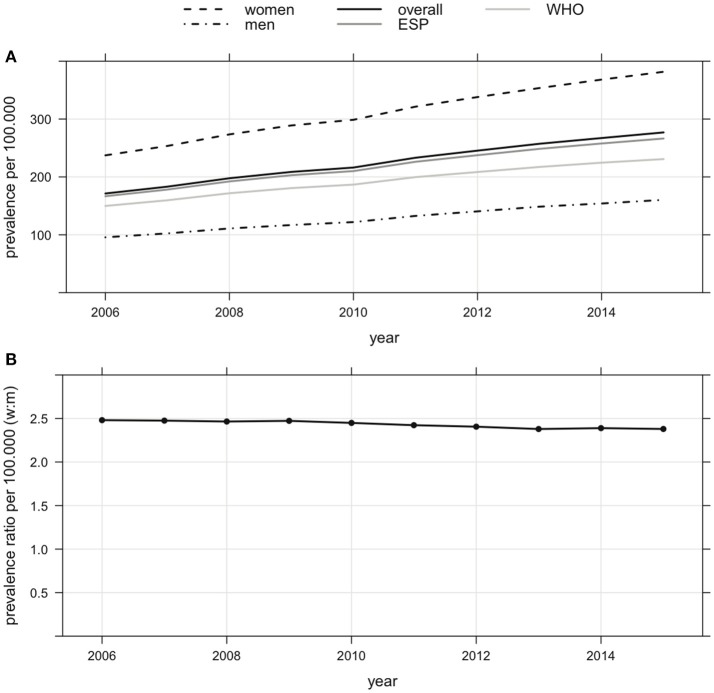
**(A)** Prevalence of MS (overall, women, men and standardized to the age distribution) of the European standard population (ESP) and the WHO standard population in Bavaria from 2006 to 2015 (increase of overall prevalence of MS by 62% from 171 per 100,000 in 2006 to 277 per 100,000 in 2015, increase of prevalence of MS of men by 68% from 96 per 100,000 in 2006 to 160 per 100,000 in 2015, increase of prevalence of MS of women by 61% from 237 per 100,000 in 2006 to 382 per 100,000 in 2015). **(B)** Female to male ratio of prevalence of MS in Bavaria from 2006 to 2015 (decrease from 2.48 in 2006 to 2.38 in 2015).

The overall prevalence of MS in Bavaria increased by 62%, from 171 per 100,000 in 2006 to 277 per 100,000 in 2015 (Figure 1A, Table 1). The prevalence standardized to the age distribution of the European standard population and the WHO standard population are also given in Figure 1A and Table 1. In 2015, about 30,000 of 10,720,000 Bavarians enrolled in statutory health insurance plans were diagnosed with MS. Assuming a similar prevalence across Germany, this would suggest that approximately 230,000 persons were diagnosed with MS in 2015. The prevalence in the male population increased by 68%, from 96 per 100,000 in 2006 to 160 per 100,000 in 2015 (Figure 1A, Table 1). The prevalence in the female population increased by 61%, from 237 per 100,000 in 2006 to 382 per 100,000 in 2015 (Figure 1A, Table 1). Thus, the female to male ratio remained largely unchanged during the observation period, equalling 2.48 in 2006 and 2.38 in 2015 (Figure 1B, Table 1).

**Table 1:** Overall Incidence and Prevalence of MS in Bavaria.

		**2006**	**2007**	**2008**	**2009**	**2010**	**2011**	**2012**	**2013**	**2014**	**2015**
Women	MS Cases	13,175	14,084	15,205	16,047	16,558	17,809	18,775	19,713	20,669	21,548
	New Cases	1344	1,251	1,361	1,247	1,258	1,243	1,296	1,338	1,338	1,221
	Population	5,552,393	5,555,248	5,559,404	5,553,047	5,542,847	5,541,188	5,554,480	5,574,694	5,613,912	5,645,713
	Prevalence	237.29	253.53	273.5	288.98	298.73	321.39	338.02	353.62	368.17	381.67
	Incidence	24.21	22.52	24.48	22.46	22.70	22.43	23.33	24.00	23.83	21.63
Men	MS cases	4,624	4,957	5,387	5,673	5,919	6,452	6,885	7,341	7,724	8,138
	New cases	499	445	536	480	512	524	539	582	548	543
	Population	4,834,814	4,840,349	4,855,989	4,854,931	4,853,063	4,864,607	4,900,891	4,939,410	5,012,757	5,074,075
	Prevalence	95.64	102.41	110.94	116.85	121.96	132.63	140.48	148.62	154.09	160.38
	Incidence	10.32	9.19	11.04	9.89	10.55	10.77	11.00	11.78	10.93	10.70
Ratio (w:m)	Prevalence	2.48	2.48	2.47	2.47	2.45	2.42	2.41	2.38	2.39	2.38
	Incidence	2.35	2.45	2.22	2.27	2.15	2.08	2.12	2.04	2.18	2.02
Total	MS Cases	17,799	19,041	20,592	21,720	22,477	24,261	25,660	27,054	28,393	29,686
	New Cases	1,843	1,696	1,897	1,727	1,770	1,767	1,835	1,920	1,886	1,764
	Population	10,387,207	10,395,597	10,415,393	10,407,978	10,395,910	10,405,795	10,455,371	10,514,104	10,626,669	10,719,788
	Prevalence	171.36	183.16	197.71	208.69	216.21	233.15	245.42	257.31	267.19	276.93
	Incidence	17.74	16.31	18.21	16.59	17.03	16.98	17.55	18.26	17.75	16.46
ESP^*^	Prevalence	166.7	178.18	192.24	202.98	210.09	226.19	237.55	248.51	257.8	266.36
	Incidence	17.14	15.85	17.66	16.19	16.67	16.56	17.2	17.91	17.38	15.98
WHO^*^	Prevalence	149.87	159.66	171.79	180.67	186.74	199.65	208.48	217.24	224.62	230.8
	Incidence	17.81	16.58	18.44	17	17.59	17.31	18.06	18.67	18.33	16.88

* *Estimates standardized to the age distribution of the European standard population (ESP) and the WHO standard population*.

**Figure 2:**
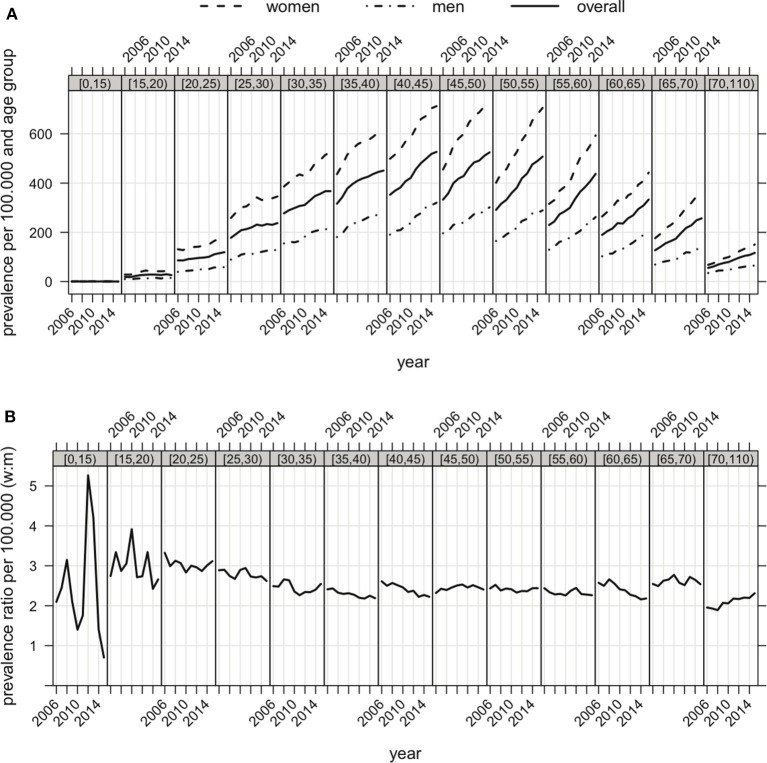
**(A)** Prevalence of MS in Bavaria (overall, women and men) correspondent to age groups (displayed in the gray bar on top of each graph) from 2006 to 2015. **(B)** Female to male ratio of prevalence of MS in Bavaria correspondent to age groups from 2006 to 2015.

The MS prevalence increased in all age groups (Figure 2A, Table 2). Overall, the median age was not different in 2006 and 2015, varying between 40 and 45 years. Therefore, 50% of the MS patients were at least 40 years. In both men and women, the strongest increase in prevalence was observed in those aged 40–60 years old. The median of the female to male ratios computed within the age groups showed only little change during the observation period, ranging from 2.49 in 2006 to 2.41 in 2015 (Figure 2B, Table 2). The female to male ratio was higher in young adults (Figure 2B, Table 2).

**Table 2:** Age dependent Incidence and Prevalence of MS in Bavaria.

**Age_group**		**2006**	**2007**	**2008**	**2009**	**2010**	**2011**	**2012**	**2013**	**2014**	**2015**
[0,15)	MS cases	9	10	12	9	14	8	12	10	7	5
	New cases	5	5	8	3	10	3	7	6	6	2
	Population	1,533,054	1,500,539	1,472,703	1,443,013	1,413,975	1,387,482	136,6197	1,352,403	1,351,951	1,360,360
	Prevalence	0.59	0.67	0.81	0.62	0.99	0.58	0.88	0.74	0.52	0.37
	Incidence	0.33	0.33	0.54	0.21	0.71	0.22	0.51	0.44	0.44	0.15
[15,20)	MS cases	119	113	142	160	165	168	165	154	171	151
	New cases	45	48	55	66	60	66	57	58	66	49
	Population	623,937	627,201	622,773	612,648	595,180	586,792	581,870	575,261	573,771	572,515
	Prevalence	19.07	18.02	22.80	26.12	27.72	28.63	28.36	26.77	29.80	26.37
	Incidence	7.21	7.65	8.83	10.77	10.08	11.25	9.80	10.08	11.50	8.56
[20,25)	MS cases	525	525	572	592	620	636	663	727	760	776
	New cases	154	142	166	159	184	155	162	173	193	180
	Population	608,642	611,967	625,598	633,598	645,438	651,909	658,682	657,356	659,323	650,837
	Prevalence	86.26	85.79	91.43	93.43	96.06	97.56	100.66	110.59	115.27	119.23
	Incidence	25.30	23.20	26.53	25.09	28.51	23.78	24.59	26.32	29.27	27.66
[25,30)	MS cases	1,127	1,242	1,362	1,394	1,424	1,500	1,499	1,587	1,626	1,738
	New cases	236	238	281	237	259	276	287	283	275	299
	Population	633,631	642,575	651,954	653,794	646,814	648,168	659,351	679,261	705,046	731,414
	Prevalence	177.86	193.28	208.91	213.22	220.16	231.42	227.34	233.64	230.62	237.62
	Incidence	37.25	37.04	43.10	36.25	40.04	42.58	43.53	41.66	39.00	40.88
[30,35)	MS cases	1,703	1,755	1,833	1,912	1,982	2,141	2,328	2,442	2,580	2,616
	New cases	261	241	234	237	232	225	285	293	281	266
	Population	614,063	604,908	614,252	622,829	637,488	650,696	669,943	685,787	702,787	712,210
	Prevalence	277.33	290.13	298.41	306.99	310.91	329.03	347.49	356.09	367.11	367.31
	Incidence	42.50	39.84	38.10	38.05	36.39	34.58	42.54	42.72	39.98	37.35
[35,40)	MS cases	2,526	2,579	2,669	2,631	2,599	2,581	2,621	2,749	2,894	3,051
	New cases	315	269	282	251	244	209	230	268	271	223
	Population	798,402	753,426	706,011	664,101	633,943	615,545	615,311	628,962	649,863	676,778
	Prevalence	316.38	342.30	378.04	396.17	409.97	419.30	425.96	437.07	445.32	450.81
	Incidence	39.45	35.70	39.94	37.80	38.49	33.95	37.38	42.61	41.70	32.95
[40,45)	MS cases	3,150	3,301	3,373	3,514	3,506	3,639	3,654	3,580	3,562	3,522
	New cases	329	257	298	283	240	272	239	238	230	210
	Population	893,483	893,272	883,202	862,462	832,488	797,190	758,790	716,758	687,119	668,637
	Prevalence	352.55	369.54	381.91	407.44	421.15	456.48	481.56	499.47	518.40	526.74
	Incidence	36.82	28.77	33.74	32.81	28.83	34.12	31.50	33.21	33.47	31.41
[45,50)	MS cases	2,652	2,948	3,401	3,641	3,786	4,104	4,334	4,379	4,504	4,522
	New cases	200	231	258	209	238	226	234	218	236	206
	Population	796,731	823,499	850,754	870,405	882,514	890,561	895,780	890,874	881,293	861,845
	Prevalence	332.86	357.98	399.76	418.31	429.00	460.83	483.82	491.54	511.07	524.69
	Incidence	25.10	28.05	30.33	24.01	26.97	25.38	26.12	24.47	26.78	23.90
[50,55)	MS cases	1,934	2,156	2,343	2,636	2,908	3,333	3,615	4,060	4,312	4,560
	New cases	139	118	133	130	130	154	142	173	145	160
	Population	663,667	681,315	705,433	732,961	763,466	794,711	823,634	853,777	880,535	898,352
	Prevalence	291.41	316.45	332.14	359.64	380.89	419.40	438.91	475.53	489.70	507.60
	Incidence	20.94	17.32	18.85	17.74	17.03	19.38	17.24	20.26	16.47	17.81
[55,60)	MS cases	1,423	1,532	1,728	1,813	1,916	2,190	2,453	2,687	2,986	3,339
	New cases	59	70	89	63	90	85	93	96	84	84
	Population	618,679	627,952	632,366	636,199	640,150	654,434	672,654	698,240	730,463	763,296
	Prevalence	230.01	243.97	273.26	284.97	299.30	334.64	364.67	384.82	408.78	437.44
	Incidence	9.54	11.15	14.07	9.90	14.06	12.99	13.83	13.75	11.50	11.00
[60,65)	MS cases	985	1,074	1,143	1,288	1,368	1,536	1,632	1,801	1,903	2,070
	New cases	40	25	34	41	41	48	40	43	37	36
	Population	519,345	524,174	529,933	542,222	580,978	597,700	606,367	610,917	616,057	621,266
	Prevalence	189.66	204.89	215.69	237.54	235.47	256.99	269.14	294.80	308.90	333.19
	Incidence	7.70	4.77	6.42	7.56	7.06	8.03	6.60	7.04	6.01	5.79
[65,70)	MS cases	858	920	972	961	909	965	1,077	1,142	1,274	1,406
	New cases	34	27	35	25	15	17	28	28	28	28
	Population	673,449	654,293	625,791	584,910	521,565	489,285	493,699	499,743	511,731	547,514
	Prevalence	127.40	140.61	155.32	164.30	174.28	197.23	218.15	228.52	248.96	256.80
	Incidence	5.05	4.13	5.59	4.27	2.88	3.47	5.67	5.60	5.47	5.11
[70,100)	MS cases	788	886	1,042	1,169	1,280	1,460	1,607	1,736	1,814	1,930
	New cases	26	25	24	23	27	31	31	43	34	21
	Population	1,410,124	1,450,476	1,494,623	1,548,836	1,601,911	1,641,322	165,3093	1,664,765	1,676,730	1,654,764
	Prevalence	55.88	61.08	69.72	75.48	79.90	88.95	97.21	104.28	108.19	116.63
	Incidence	1.84	1.72	1.61	1.48	1.69	1.89	1.88	2.58	2.03	1.27

### Incidence

The incidence of MS was investigated in the same population. Between 2006 and 2015 the incidence remained rather stable (median 17.3, range 16.3 to 18.3 per 100,000 inhabitants, Table 1). The incidence standardized to the age distribution of the European standard population and the WHO standard population are also given in this Table 1. Incidence was higher among women (median 23.0, range 21.6–24.5 per 100,000) than among men (median 10.7, range 9.2–11.8 per 100,000).

The highest incidence rates were found in persons aged 20–50 years, with a peak incidence rate of 30.3 per 100,000 in men aged 30–35 and 61.1 per 100,000 in women aged 25–30 (Table 2). Incidence rates increased slightly in the age group 15–30 but decreased slightly in older patients during the observation period.

### Regional distribution

**Figure 3:**
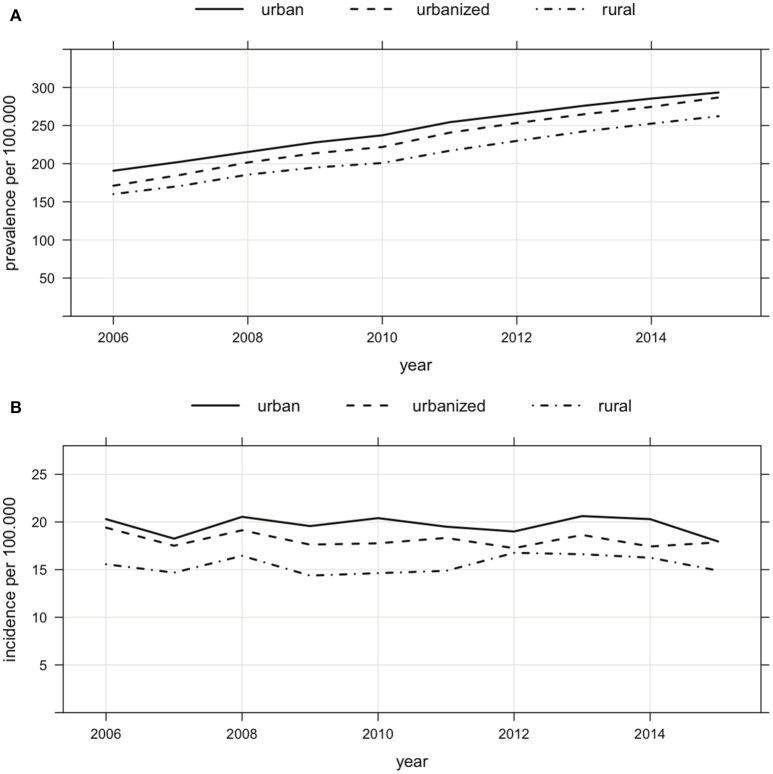
**(A)** Regional distribution of prevalence of MS in Bavaria (urban, urbanized and rural areas) from 2006 to 2015 (prevalence of 293 in urban, of 287 in urbanized and of 262 per 100,000 in rural areas in 2015, increase of prevalence from 2006 to 2015 in urban by 54%, urbanized by 68% and rural by 64%). **(B)** Regional distribution of incidence of MS in Bavaria (urban, urbanized and rural areas) from 2006 to 2015.

The prevalence was higher in urban areas compared to partially urbanized and rural areas. In 2015, the prevalence was 293 in urban, 287 in partially urbanized and 262 per 100,000 in rural areas (Figure 3A). Between 2006 and 2015, the prevalence increased in urban areas by 54%, in urbanized areas by 68% and in rural areas by 64%. Incidence rates were higher in urban areas as compared to partially urbanized or rural areas (Figure 3B).

## Future prevalence rates

**Figure 4:**
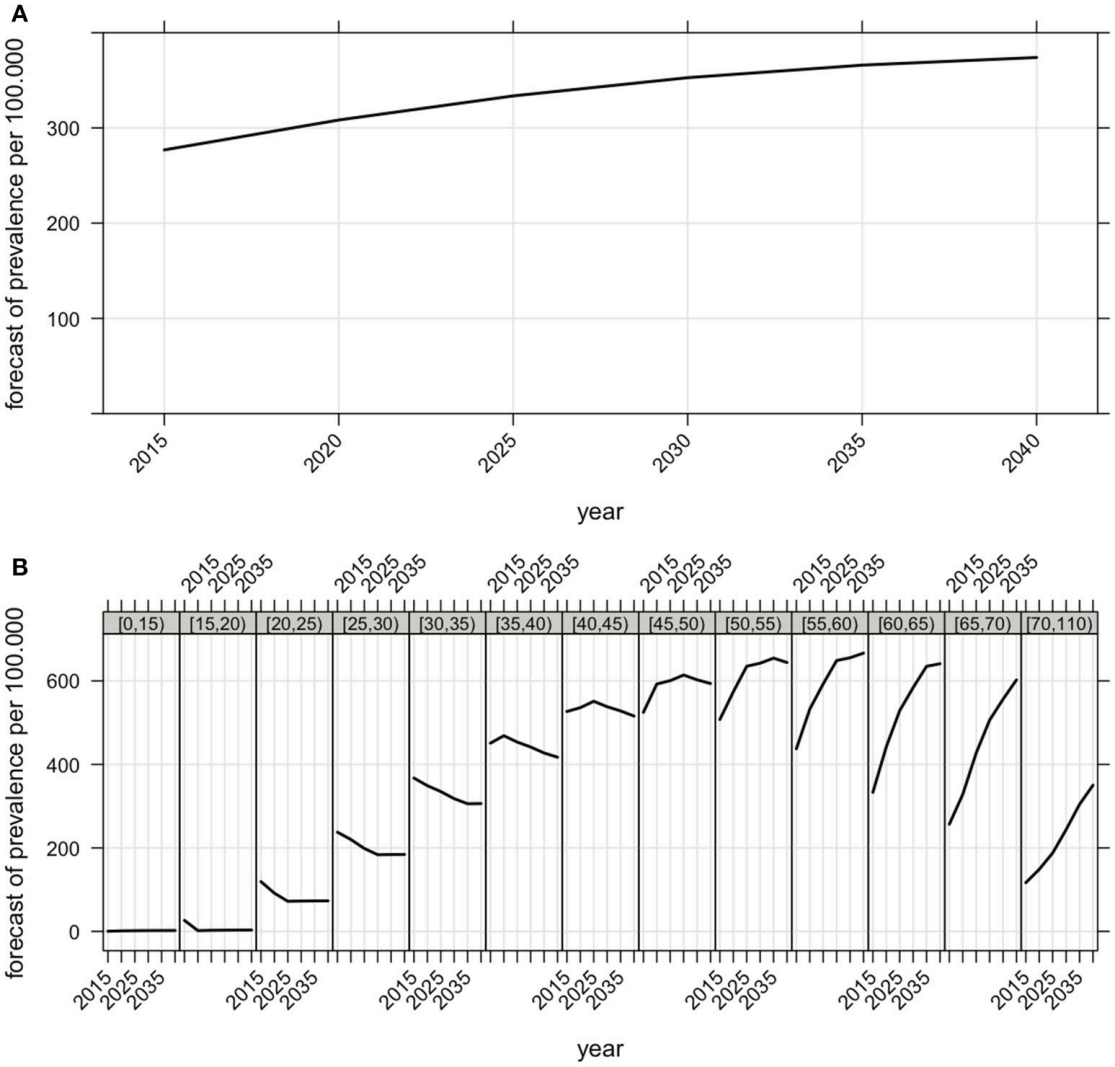
**(A)** Forecast of the future overall prevalence of MS in Bavaria (the prevalence of 277 per 100,000 inhabitants in 2015 is expected to increase to 353 per 100,000 inhabitants by 2030 and to 374 per 100,000 inhabitants by 2040). **(B)** Forecast of the future prevalence of MS correspondent to age groups in Bavaria. Computational details are given in the methods section.

The future prevalence rates in Bavaria were estimated as outlined in the methods section. According to our predictive model, the prevalence is set to reach 353 per 100,000 inhabitants by 2030 and 374 per 100,000 inhabitants by 2040, with 50% of the MS patients being older than 45 years in 2040 (Figure 4A). While a small decrease in prevalence is expected in patients below the age of 45, a sharp increase is expected among older people. In 2040, the highest prevalence will be observed in the people aged between 50 and 65 years (Figure 4B).

## Discussion

Multiple sclerosis is one of the leading causes of disability in young adults. Besides individual implications for the affected people, the disease represents a significant socioeconomic burden to society. We present the first authoritative data on the prevalence and incidence of MS in Bavaria, one of the largest states in Germany with more than 12 million inhabitants. We observed a notable increase in the number of MS patients over the last decade, with 18,000 Bavarians enrolled in public health insurance plans being affected in 2006 and 30,000 in 2015. This corresponds to a 62% increase in the overall prevalence, reaching 277 MS patients per 100,000 Bavarians in 2015. Assuming the same prevalence in Germany as a whole, this would suggest that currently more than 230,000 Germans are diagnosed with MS. As the prevalence among women increased at the same rate as among men between 2006 and 2015, the female to male ratio remained largely unchanged. There was no trend in the incidence rates over the last decade, which ranged from 16 to 18 per 100,000 people. We observed a slightly higher prevalence and incidence of MS in urban areas as compared to partially urbanized and rural areas. According to our forecast, the prevalence of MS will further increase at least until 2040, when we predict a prevalence of almost 374 per 100,000 Bavarians, with more than 50% of MS patients being older than 45 years. Assuming a similar trend across Germany, this would translate into more than 300,000 Germans diagnosed with MS in 2040. A sharp increase of prevalence is expected for people aged between 55 and 70 years while a small decrease in prevalence is expected in patients below the age of 45.

Previous studies of prevalence and incidence have applied different methodological approaches and can be affected by inaccuracy of diagnosis and data ascertainment with subsequently restricted comparability. A study by Hoer A and coworkers analyzed the prevalence of MS in Bavaria from 2005 to 2009 using the same ambulatory claims data combined with prescribing data (10). The study took a different approach to identifying MS patients, requiring either a single diagnosis from a neurologist or psychiatrist or the collection of MS-specific medication from a pharmacy (10). It found that MS prevalence increased from 0.123 to 0.175%, which was then projected to 102,000 to 143,000 patients in the whole German population (10). However, patient identification was based solely on a health insurance number that was subject to change (e.g., marriage, change of status or change of insurance company). The use of a persistent patient identifier enables the present study to observe patients reliably over the entire observation period, improving estimation of prevalence and enabling the estimation of incidence.

Another study evaluated nationwide Statutory Health Insurance data covering both inpatient and outpatient services in Germany for the year 2010 counting approximately 200,000 patients with MS diagnosis (13). An investigation by Hein and coworkers used another approach with data based on representative samples of groups of physicians engaged in MS treatment, with the methodological risk of double counting and without the possibility of questioning the diagnosis (12).

In the present study we performed a systematic retrospective analysis of anonymous data held by the *Bavarian Association of Statutory Health Insurance Physicians* covering 83.5% of the Bavarian population between 2004 and 2016. In this respect the data of MS incidence, prevalence and regional distribution corresponded to a great majority of the Bavarian population. The data represent the claims of outpatient health care providers (outpatient clinics, general practitioners and registered medical specialists) and contains broad and objective information related to MS patients. The data include regional information, allowing stratification not only by age group and gender, but also by regional aspects. A minimum observation period of 2.5 years was used to rule out any prior MS diagnoses. We provide evidence to show that the prevalence in Bavaria is among the highest in the world, reaching 277 MS patients per 100,000 people in 2015.

Studies from other countries reported similar prevalence rates such as Canada (240 per 100,000 in 2000/2001), Northern Ireland (200.5 per 100,000 in 1996) and Scotland (between 203 and 219 per 100,000) (19–21). Older studies from central European countries like Hungary (62–65 per 100,000 people at the end of the 1990s), Austria (98.5 per 100,000 at the end of the 1990s) and Switzerland (110 per 100,000 in 1986) reported lower prevalence rates (22–25). We observed an increased prevalence over the past decade similar to the results reported by studies from Denmark (58.8 in 1950 to 154.5 per 100,000 in 2005) and Norway (19.3 in 1980 to 182.4 per 100,000 in 2010) (4, 26, 27).

Several investigations provide evidence for an increasing incidence of MS (e.g., in northern Finland with an increase especially in women from 1992 to 2007 and in the Nordland County of Norway from 0.7 per 100,000 in 1970 to 1974 to 10.1 per 100,000 in 2005 to 2009) (27, 28). In our study, we found that incidence rates were stable in Bavaria between 2006 and 2015. This is one of the surprising findings of our study with an increase in prevalence despite stable incidence rates during the observation period. This might be due to an increase in incidence before the observation period, possibly caused by the broad availability of magnetic resonance imaging and introduction of new diagnostic criteria in 1983 and 2001 (29, 30). The access to more and more effective immunotherapies may have improved life expectancy of affected patients and thus have increased MS prevalence despite stable incidence rates (4, 31).

Focusing on the female to male ratio of prevalence and incidence of MS, meta-analyses of studies over the previous 20-30 years provide evidence of a general but not ubiquitous increase in the female to male ratio of incidence of MS possibly due to changes in lifestyle, particularly among women (8, 32). In the present study, the prevalence increased at the same rate in the male and female populations between 2006 to 2015.

Looking at the regional distribution, our study found that urban areas were associated with a higher prevalence than partially urbanized and rural areas. Moreover, incidence rates were higher in urban as compared to partially urbanized and rural areas. These results are in line with data of studies of Finland, Canada and South America that show regional differences in MS prevalence and incidence (19, 32–34). These differences might be explained by a better access of patients to health care providers in urbanized and urban areas including neurological doctor‘s offices as well as radiologists with MRI scanners (19, 31–36). Also environmental factors might contribute to regional differences (32–34, 37).

In this study, data on ethnic differences in the development of MS prevalence and incidence rates in the last years was not available but would be of great interest for future studies.

Our study has certain limitations. The diagnosis of MS is based on the assessment of individual Bavarian neurologists in private practice. In this respect, the diagnostic MS criteria on which clinicians based their diagnosis are unknown. Because physicians in private practice are required to participate in continuing education training and many activities for neurologists are focused on MS, we assume a high standard of MS care in private practice including knowledge on the current diagnostic MS criteria.

Patients undiagnosed in the preliminary stages of the disease and otherwise unreported cases are unaccounted for, possible leading to an underestimation of the prevalence of MS in Bavaria. Another reason for an underestimation of prevalence could be incorrect coding by physicians. Most frequently, physicians used non-specified MS diagnosis codes (ICD-10 code G35.9), hence there is only partial information on the frequency of different types and courses of MS in Bavaria. The study data encompassed 83.5% of the total Bavarian population (see Methods), limiting generalizability to the population as a whole. Moreover, the data do not cover the treatment of patients in hospitals or in outpatient units of university hospitals. Furthermore another diversification of the study might be a possible difference of the Bavarian population compared to the overall German population in respect to demographic characteristics. We counteracted this fact by using age-group- and gender-specific measures for a projection of the results onto the entire German population and standardized our results to the age distribution of the European standard population (ESP) and the WHO standard population. However we cannot rule out further, unmeasured differences in the demographic and regional structure.

In summary, our results on population based insurance data depict a considerable increase of MS prevalence in Bavaria from 2006 to 2015. The rise of prevalence of MS demonstrates a need to strengthen healthcare provision systems due to the increasing numbers of particularly older patients with MS in the next years. Future research should extend these data to the entirety of Germany. Precise information about the distribution of MS in Germany is necessary to inform the capacity planning of health care services from an economic perspective as well as from the perspective of those affected by MS.

## Ethics statement

For this retrospective study anonymous claims data of people insured by the Association of Statutory Health Insurance Physicians of Bavaria from 2006 to 2015 were analyzed. Official statistics of the German Federal Ministry of Health were used to provide the size of the general population. An ethic votum was therefore not needed.

## Author contributions

All authors listed have made a substantial, direct and intellectual contribution to the work, and approved it for publication.

### Conflict of interest statement

BH has served on scientific advisory boards for F. Hoffmann-La Roche Ltd., Novartis, Bayer AG, and Genentech; he has served as DMSC member for AllergyCare and TG therapeutics; he or his institution have received speaker honoraria from Biogen Idec, Teva Neuroscience, Merck Serono, Medimmune, Novartis, Desitin, and F. Hoffmann-La Roche Ltd.; his institution has received research support from Chugai Pharmaceuticals and Biogen; holds part of two patents; one for the detection of antibodies against KIR4.1 in a subpopulation of MS patients and one for genetic determinants of neutralizing antibodies to interferon β. AH: Received a honorarium from Biogen for consulting services for the Biogen Symposium on Statistical Methods in Real World Evidence 2017. The remaining authors declare that the research was conducted in the absence of any commercial or financial relationships that could be construed as a potential conflict of interest.
